# Changes in beta-catenin expression and activation during progression of primary sclerosing cholangitis predict disease recurrence

**DOI:** 10.1038/s41598-021-04358-6

**Published:** 2022-01-07

**Authors:** Mary Ayers, Silvia Liu, Aatur D. Singhi, Karis Kosar, Pamela Cornuet, Kari Nejak-Bowen

**Affiliations:** 1 Children’s Hospital of Pittsburgh, Pittsburgh, PA USA; 2 Department of Pathology, School of Medicine, University of Pittsburgh, S405A-BST, 200 Lothrop Street, Pittsburgh, PA 15261 USA; 3 Pittsburgh Liver Research Center, University of Pittsburgh, Pittsburgh, PA USA

**Keywords:** Biliary tract disease, Liver, Prognostic markers

## Abstract

Primary sclerosing cholangitis (PSC) is a rare, chronic, cholestatic liver disease characterized by progressive inflammation and fibrosis of the bile ducts. We have previously demonstrated the importance of Wnt/β-catenin signaling in mouse models of PSC. In this study, we wished to determine the clinical relevance of β-catenin localization in patient samples. In livers explanted from patients diagnosed with PSC, the majority (12/16; 75%) lacked β-catenin protein expression. Biopsies from patients post-transplant were classified as recurrent or non-recurrent based on pathology reports and then scored for β-catenin activation as a function of immunohistochemical localization. Despite lack of statistical significance, patients with recurrent primary disease (n = 11) had a greater percentage of samples with nuclear, transcriptionally active β-catenin (average 58.8%) than those with no recurrence (n = 10; 40.53%), while non-recurrence is correlated with β-catenin staining at the cell surface (average 52.63% for non-recurrent vs. 27.34% for recurrent), as determined by three different methods of analysis. β-catenin score and years-to-endpoint are both strongly associated with recurrence status (*p* = 0.017 and *p* = 0.00063, respectively). Finally, there was significant association between higher β-catenin score and increased alkaline phosphatase, a marker of biliary injury and disease progression. Thus, β-catenin expression and activation changes during the progression of PSC, and its localization may be a useful prognostic tool for predicting recurrence of this disease.

## Introduction

Primary sclerosing cholangitis (PSC) is a chronic, progressive, cholestatic liver disease that affects the intrahepatic and/or extrahepatic bile ducts. Continuous inflammation of the ducts causes concentric (“onion-skin”) periductal fibrosis, which eventually leads to obliteration and loss of bile ducts and the formation of a fibrous scar^1,2^. Biliary fibrosis obstructs bile flow, which results in accumulation of “toxic bile” in the liver; this is thought to be a major contributor to the pathogenesis of this disease^3^.

There is no FDA-approved disease-altering treatment for PSC, and the only life extending treatment is liver transplantation. Almost half of patients with PSC will experience disease progression, with end stage liver disease or cancer requiring transplant. Despite transplant, the disease will recur in about 25% of patients^4^. Risk factors for recurrent PSC include co-morbidities such as inflammatory bowel disease or cholangiocarcinoma^5^; however, there are currently no specific biochemical parameters that predict clinical outcome after transplantation. Currently, elevated levels of alkaline phosphatase (ALP), a serum marker of biliary injury, are associated with greater risk of negative outcomes, such as liver transplantation and death^6^. However, serum ALP levels tend to fluctuate over time due to transient blockage of strictured ducts and this may limit its usefulness as a surrogate marker for recurrence^7^. Histological staging of liver fibrosis in biopsies has also been used to predict whether patients will progress over time^8^, but this procedure is not favored due to its invasive nature and sampling variability^7^. Clearly, identification of biomarkers that help to predict which patients might develop recurrent PSC would have important clinical implications.

β-catenin is the effector of the Wnt signaling pathway, which has a well-described yet multifaceted role in liver physiology and pathology. Its normal activity is essential for hepatic development, biliary morphogenesis, postnatal hepatic growth, and hepatocyte maturation (reviewed in^9^). Conversely, β-catenin activation also promotes the development and progression of liver diseases such as diet-induced steatohepatitis, fibrosis, and hepatocellular cancer (reviewed in^10^). β-catenin is expressed throughout the adult liver, and can be found at the cell membrane, where it exists as a part of adherens junctions, as well as inside the cell, where it is normally degraded in the absence of Wnts. Activation of this pathway occurs when Wnt protein binds to its receptor Frizzled and co-receptor low density lipoprotein–related protein 5 or 6 on the cell surface, resulting in a cascade of events that frees β-catenin from destruction, allowing it to accumulate in the cytoplasm and eventually translocate to the nucleus, where it activates target gene expression. In the normal adult liver, nuclear localization of β-catenin is found only in the pericentral hepatocytes, where it regulates metabolic zonation^10^. However, in pathological conditions such as hepatocellular carcinoma (HCC), β-catenin can become constitutively activated and translocate to the nucleus irrespective of location within the hepatic lobule.

In addition to its roles in zonation and injury repair, this signaling pathway has recently been shown to alter bile acid metabolism as well as the progression of cholestatic liver disease in animal models of PSC^11^. Cytochrome P450 (Cyp)7a1, the rate limiting enzyme in the synthesis of bile acids from cholesterol, is expressed in the pericentral zone of the liver, coincident with constitutive activation of β-catenin^12^. Furthermore, Cyp7a1 is suppressed in the absence of β-catenin, suggesting that this gene is either a direct or indirect target of Wnt signaling^13,14^. β-catenin also forms an inhibitory complex with farnesoid X receptor (FXR), the bile acid nuclear receptor that represses Cyp7a1. Loss of β-catenin from hepatocytes eliminates sequestration of FXR, which decreases bile acid synthesis and lowers the level of total hepatic bile acids, resulting in protection from bile duct ligation-induced liver injury, fibrosis, and ductular response^13^. That loss of β-catenin is associated with a protective phenotype suggests that alterations in β-catenin expression could have predictive and perhaps therapeutic implications for human cholestatic liver disease.

To determine if these findings were relevant to PSC, we measured the expression of β-catenin protein in explanted livers from patients transplanted for PSC, as well as in post-transplant biopsies from PSC patients. The results indicate that β-catenin expression and/or localization is not only a useful marker of disease progression but is also a prognostic marker for disease recurrence.

## Methods

### Patient tissues

All experimental protocols were approved by the University of Pittsburgh Institutional Review Board (IRB) Office of Research Protections, and all methods were carried out in accordance with relevant guidelines and regulations. All tissues and materials used in this retrospective study were obtained under the IRB protocols listed below, and informed consent was waived by the University of Pittsburgh IRB.

Frozen liver samples from 16 patients with a primary diagnosis of PSC at the time of explant, as well as liver samples from 7 control non-cholestatic patients, were obtained from the Pittsburgh Liver Research Center’s Clinical Biospecimen Repository and Processing Core (CBRPC; IRB approval number: STUDY20010114) at the University of Pittsburgh, School of Medicine. The corresponding anonymous pathology reports were also obtained, and pertinent patient information is detailed in Supplemental Table 1.

**Figure 1:**
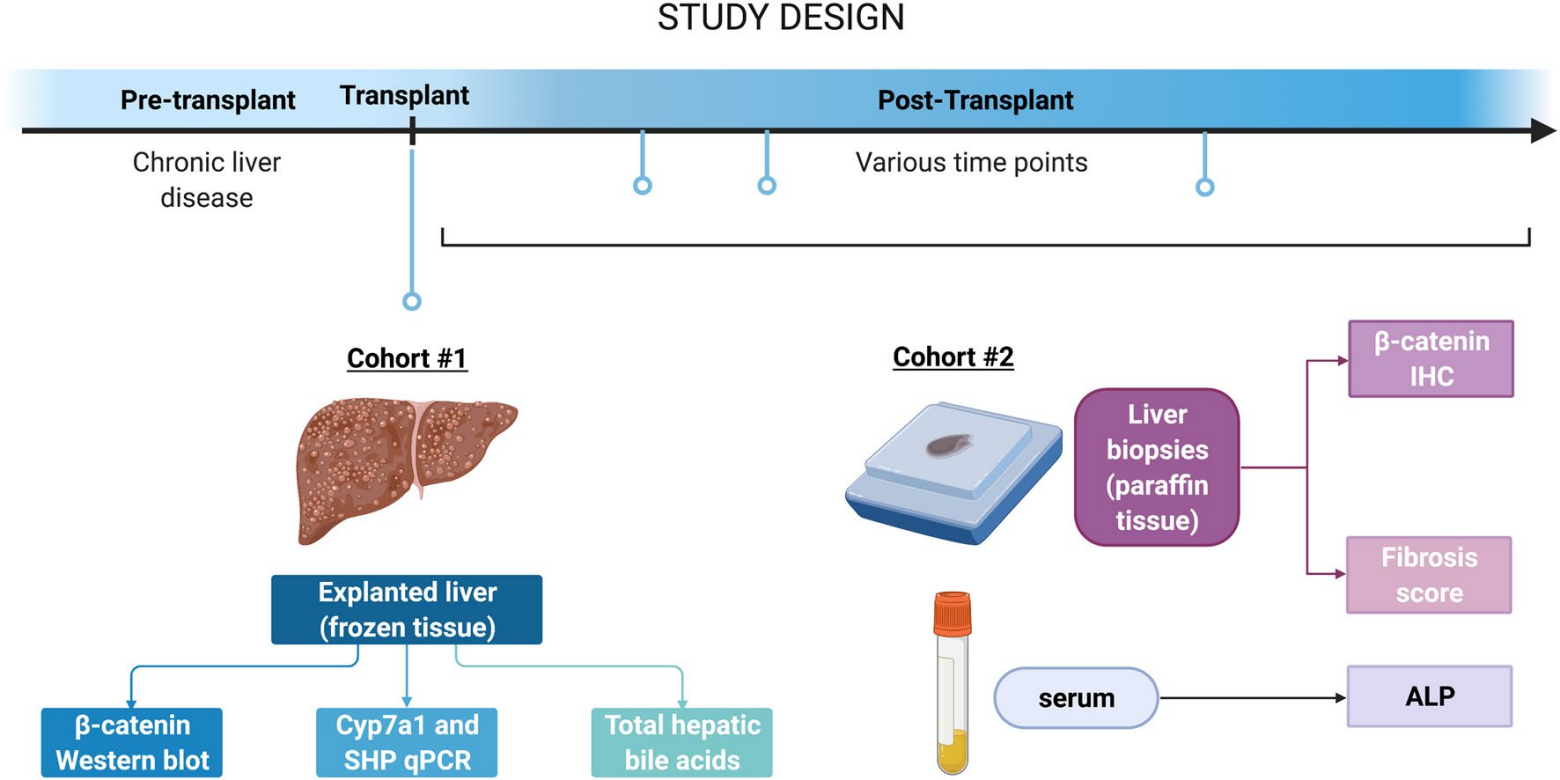
Flow chart of study design. The first cohort (n = 7 normal; n = 18 PSC) consisted of native liver removed at the time of transplant. The second cohort (n = 11 recurrent; n = 10 non-recurrent) consisted of follow-up biopsies (n ≥ 1) from livers post-transplant. Note that the two cohorts are independent of one another, and no information was available regarding patient overlap between the two groups, if any.

For the biopsy studies, a database containing a list of patients in which the primary diagnosis is PSC and who have received orthotopic liver transplantation between 1984 and 2013 was obtained. Each patient in the database had n ≥ 1 post-transplant follow-up biopsies. Based on the pathology diagnosis that accompanied each biopsy, this database was stratified into patients with non-recurrent (non-specific change, steatohepatitis, or other) and recurrent (obstruction/cholangitis or recurrent primary disease as primary diagnosis) disease. From these categories, n = 11 recurrent and n = 10 non-recurrent patients were randomly chosen for analysis. Biopsy tissues in paraffin were obtained from the Department of Pathology, School of Medicine, University of Pittsburgh (IRB approval number: STUDY19010210). The pertinent patient information and corresponding anonymous pathology reports are detailed in Supplemental Table 2. A flow chart summarizing the analyses performed on both cohorts is shown in Fig. 1.

### Protein extraction and Western blot (WB) analysis

Approximately 20 mg of tissue from explanted livers was extracted, resolved on Tris–HCl precast gels by SDS-PAGE, and transferred to polyvinylidene difluoride membranes as described previously^15^. Membranes were probed with β-catenin (1:1000, 610,154) from BD Biosciences, San Jose, CA; and glyceraldehyde-3-phosphate dehydrogenase (GAPDH; 1:400, sc-25778) from Santa Cruz Biotechnology, Dallas, TX. Densitometry analysis of WBs was performed using ImageJ software (version 1.44o). A total of 4 WBs were run in order to assess the 7 normal and 16 PSC livers. Samples MA102, MA103, MA104, and C268, F138, G139, G336, and B805 were repeated on multiple blots, and the β-catenin/GAPDH ratios were normalized to the representative blot in order to maintain consistency between blots.

### RNA isolation and real-time quantitative (q) PCR

RNA was extracted from frozen human livers, converted to cDNA, and assayed on the Applied Biosystems StepOnePlus Real-Time PCR System with StepOne v2.1 software. The comparative ΔΔCT method was used for analysis of the data and all data is presented normalized to NL at baseline. GAPDH expression was used as the internal control. The following primers were used: Cyp7a1-fwd: 5′-GGT GTT GTG CCA CGG AAA AT-3′; Cyp7a1-rev: 5′-TCC ATC CAT CGG GTC AAT GC-3′; SHP-fwd: 5′-GTG GCT TCA ATG CTG TCT GGA- ‘3; SHP-rev: 5′-ACT TCA CAC AGC ACC CAG TGA-3′; GAPDH-fwd: 5′-TCC AAA ATC AAG TGG GGC GA-3′; GAPDH-rev: 5′-TGA TGA CCC TTT TGG CTC CC-3′. For this assay, n = 16 PSC livers and n = 5 normal livers were analyzed due to insufficient sample quantity in 2 normal livers, which were used only for WB.

### Liver bile acids

Liver total bile acids were measured using a total bile acids kit from Crystal Chem (Downers Grove, IL), as per the manufacturer’s instructions. Total bile acid levels were normalized to protein concentration for each sample. For this assay, n = 16 PSC livers and n = 5 normal livers were analyzed due to insufficient sample quantity in 2 normal livers, which were used only for WB.

### Immunohistochemistry (IHC)

Paraffin sections from post-transplant biopsies were used for β-catenin IHC staining, which was performed by the BRPC. Tissue scoring was performed by an experienced pathologist (ADS) who was blinded to patient demographics and outcomes. β-catenin staining was categorized as 0, representing no stain/decreasing stain; 1, representing membranous β-catenin staining; or 2, representing membranous β-catenin with some weak nuclear staining, according to a similar classification published previously^16^. A sample was considered positive for nuclear β-catenin when at least 10 positive hepatocyte nuclei were identified in a 200 × field.

Fibrosis scores were based on pathology reports for the amount of portal fibrosis and were scored as follows: none (0); mild (1); moderate (2); severe (3). In the few cases where classification was either mild-moderate or moderate-severe, the higher score was used.

### Statistical analysis

For analysis of frozen liver samples, statistical analysis was performed with Prism version 7.0a (GraphPad Software), and comparisons between normal and PSC were deemed statistically significant by unpaired two-tailed Student’s *t* test if *p* < 0.05. For analysis of post-transplant biopsies, β-catenin score distribution across recurrence and non-recurrence status was calculated. To check the association between these two variables, contingency tables were created and Fisher’s exact test was performed.

To check the predictive value of β-catenin score and years-to-endpoint on the final recurrence status, a binomial logistic regression model was fitted. The β-catenin score served as the categorical variable (0 for decreasing membranous/no staining; 1 for membranous; and 2 for membranous/weak nuclear) and years-to-endpoint was the continuous predictor variable. Binarized recurrence status (0 for non-recurrence and 1 for recurrence) was the outcome of the regression model. Once the coefficients were estimated, ANOVA test was performed on coefficient of β-catenin score, years-to-endpoint and the interaction between score and years-to-endpoint. The statistical figures and analysis were performed by R programming via publicly available packages. Regression model and ANOVA test was performed with default parameter settings.

To test the association between β-catenin score and ALP, Wilcoxon Rank Sum test was applied, where p-values were calculated to test the difference in ALP between pairwise β-catenin scores. In addition, Fisher’s Exact test was applied to test the association between β-catenin score and fibrosis stage.

## Results

### End-stage PSC is characterized by loss of β-catenin protein and decreased bile acid synthesis

**Figure 2:**
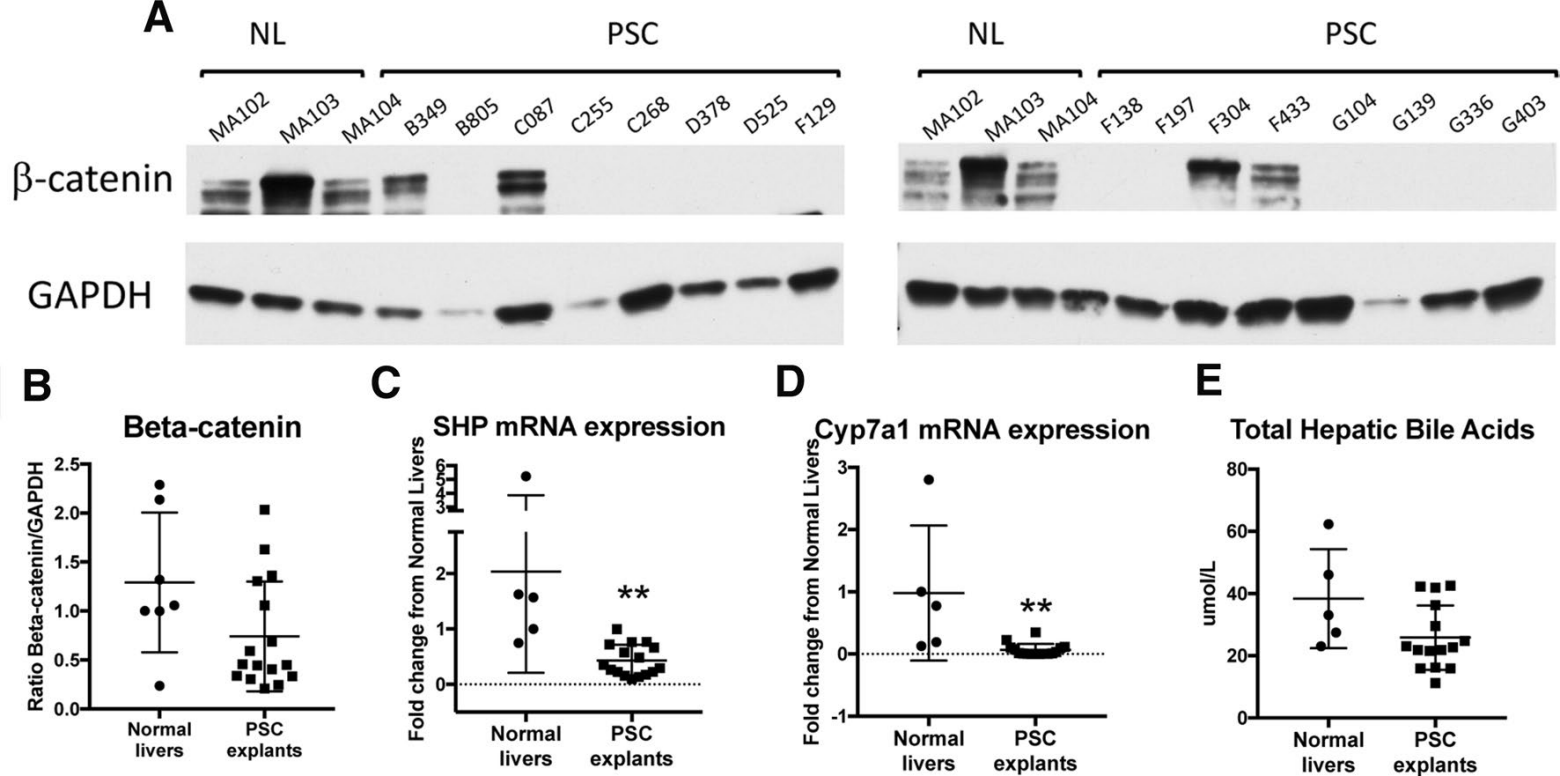
β-catenin is absent in a majority of explanted livers from PSC patients and correlates with decreased Cyp7a1 mRNA expression. (**A**) Representative WB of 16 livers explanted at the time of transplant for PSC shows loss of β-catenin in the majority of patient samples. β-catenin bands were standardized to GAPDH to control for the possibility of protein degradation due to disease. (**B**) Quantification of WBs by densitometry confirms that total β-catenin protein is decreased in most PSC samples compared to NL. (**C**) Expression of SHP mRNA decreases in livers explanted for PSC compared to NL. (**D**) Cyp7a1 mRNA expression is significantly decreased in PSC explants. (**E**) Levels of hepatic bile are unchanged between NL and PSC explants. **p < 0.01. For A and B, n = 7 NL, while for C-E, n = 5 NL due to insufficient quantity of sample.

Given our previous findings that loss of β-catenin allowed mouse livers to better adapt to chronic cholestatic injury^13^, we wanted to see if the same was true in human disease as well. Explanted liver tissue were obtained from sixteen patients with a primary diagnosis of PSC at the time of transplant, as well as from seven patients with non-cholestatic livers, and analyzed for β-catenin by Western blotting. Because advanced PSC causes liver fibrosis and can result in extensive loss of parenchyma, we standardized β-catenin expression to the expression of housekeeping gene GAPDH for accurate comparison. A representative Western blot is shown in Fig. 2A. Notably, in twelve out of sixteen PSC explanted livers β-catenin protein was entirely absent (Fig. 2A,B). The other four PSC samples had β-catenin levels that were similar to that of normal livers (NL), and did not differ in terms of demographics (age, sex) or histological description from the twelve samples lacking β-catenin.

Suppression of β-catenin results in increased expression of small heterodimer partner (SHP) mRNA, a target of FXR signaling that negatively regulates Cyp7a1^13^. However, SHP expression is not increased in PSC livers compared to NL, indicating lack of FXR activation in late-stage PSC (Fig. 2C). β-catenin is also thought to either directly or indirectly regulate transcription of Cyp7a1, the rate-limiting step in bile acid synthesis^13,14^. Therefore, we evaluated Cyp7a1 mRNA expression in PSC and NL by qPCR. Like with β-catenin, a significant decrease in Cyp7a1 expression was noted in the PSC patient samples, as shown in Fig. 2D. To determine if Cyp7a1 has any impact on bile acid synthesis, we next analyzed levels of total hepatic bile acids in liver extracts from PSC and NL explants. Interestingly, PSC livers have equivalent levels of hepatic BA compared to normal livers despite frank cholestasis, suggesting that lower Cyp7a1 helps keep bile acids in the liver to a minimum (Fig. 2E). Thus, during end-stage liver disease, spontaneous suppression of β-catenin may be one way for the liver to downregulate bile acid synthesis.

### Nuclear β-catenin localization in patient biopsies predicts disease recurrence

Given that β-catenin is absent in a majority of PSC livers that were analyzed at the time of orthotopic liver transplantation (OLT), we then wondered if β-catenin status may be a useful predictor for disease progression in PSC patients. To address this, we analyzed β-catenin expression in serial biopsies from a separate cohort of PSC patients that had received OLT. Follow-up needle biopsies are routinely done on PSC patients after transplant, and can be used to detect recurrent PSC, which occurs in 20–25% of recipient livers^17,18^. We obtained a database containing a list of patients who received OLT for PSC and had n ≥ 1 post-transplant follow-up biopsies. This database was then stratified into patients with non-recurrent or recurrent disease as described in the Methods. β-catenin staining was performed on randomly selected patients from each group (n = 11 recurrent and n = 10 non-recurrent; n ≥ 1 biopsy per patient), and localization was categorized as absent (0), membranous only (1), or membranous/weak nuclear localization (2) as described in the “Methods”.

In a normal liver, β-catenin is usually expressed at the hepatocyte membrane. However, the presence of nuclear β-catenin in hepatocytes indicates that this pathway is transcriptionally active^19^. Non-mutated nuclear β-catenin can be difficult to detect by IHC, as it is lesser in quantity and intensity than in hepatic tumors with *CTNNB1* mutations^16^. Therefore, biopsies with n > 10 positive hepatocyte nuclei per 200 × field were considered to have active β-catenin. Although PSC is a biliary disease, and β-catenin is strongly positive in cholangiocytes as well as in other non-parenchymal cells, we focused on hepatocyte localization because the role of activated β-catenin in cholangiocytes is unknown, whereas β-catenin activation in hepatocytes plays a variety of critical functions in cholestatic liver disease, including regulating bile acid metabolism and hepatocyte proliferation.

**Figure 3:**
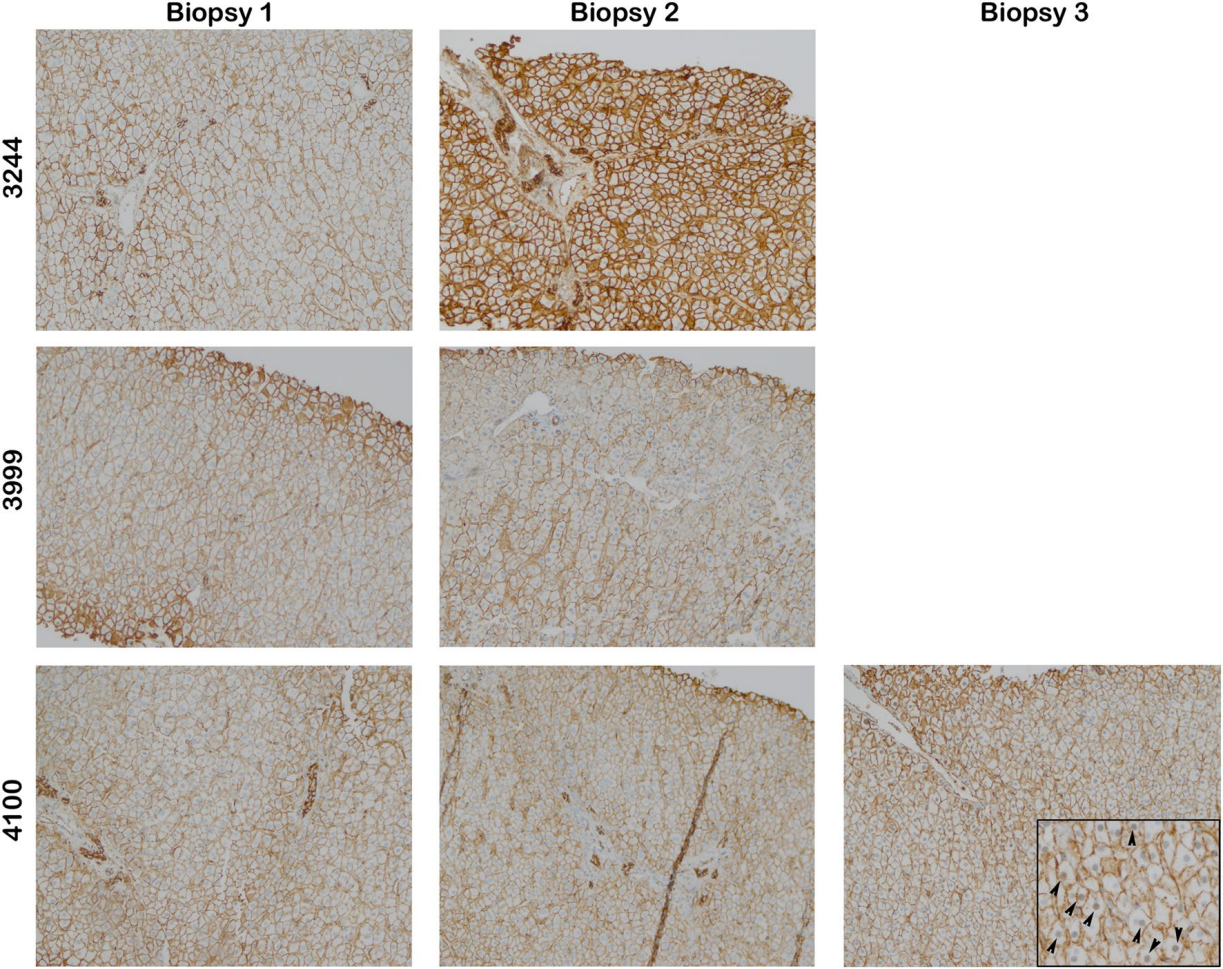
β-catenin is mostly membranous in biopsies from non-recurrent PSC patients after transplant. Representative images of post-transplant biopsies from patients classified as non-recurrent (magnification 200 × ; inset 400 ×). The majority of samples have either absent or membranous β-catenin staining only.

**Figure 4:**
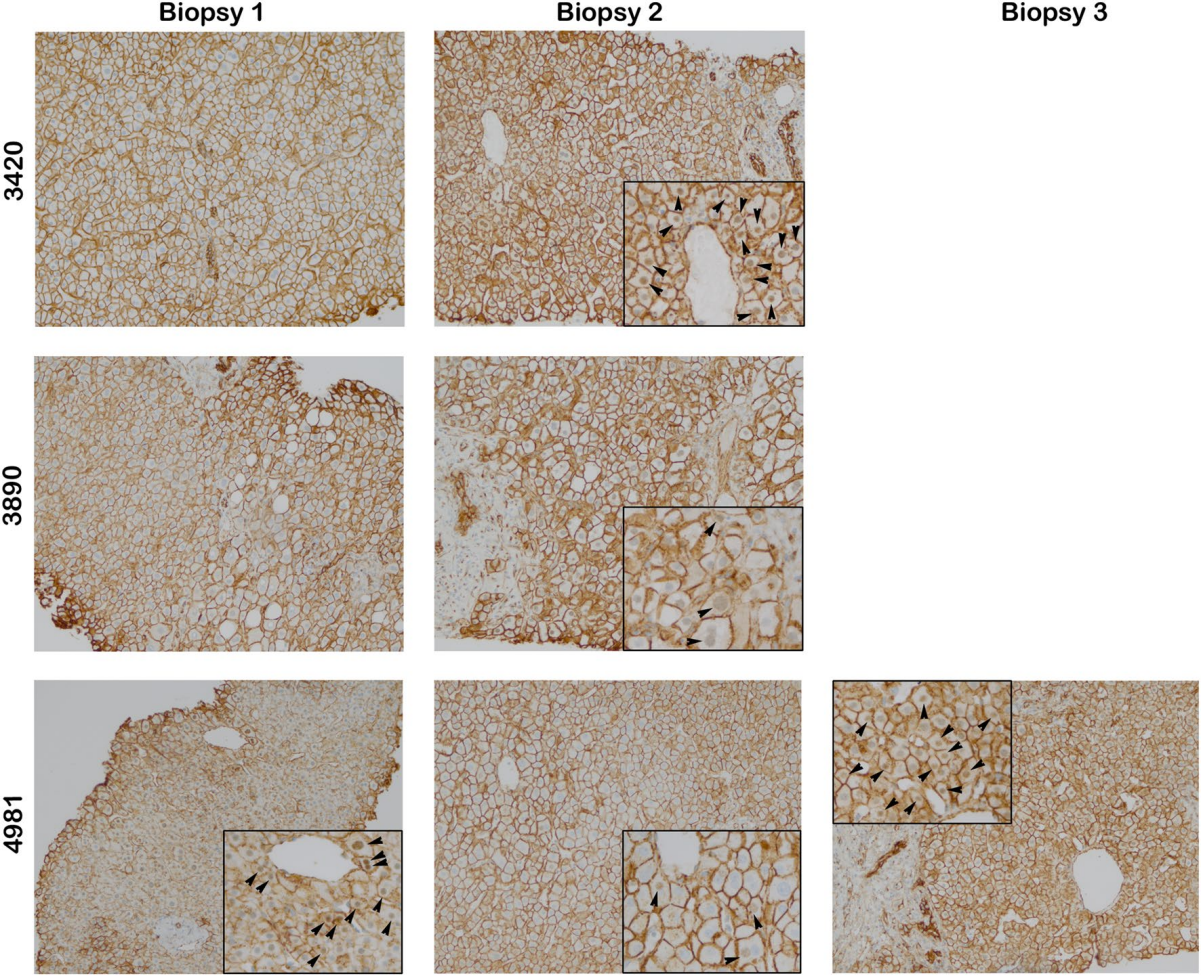
Nuclear β-catenin is present in a majority of recurrent PSC patients after transplant. Representative images of post-transplant biopsies from patients classified as recurrent for PSC (magnification 200 × ; inset 400 ×). Over half of the samples show nuclear β-catenin localization in addition to membranous staining.

β-catenin IHC from three representative non-recurrent patients is shown in Fig. 3. The first two patients (3244 and 3999) had two serial biopsies; in both, β-catenin was exclusively membranous. The third patient, 4100, had two biopsies with membranous β-catenin, while in the third multiple positive nuclei were detected (inset). Representative biopsies from patients classified with recurrent PSC are shown in Fig. 4. In these patients, nuclear β-catenin was more common, occurring in either the final biopsy (3420 and 3890) or in several consecutive biopsies (4981).

**Figure 5:**
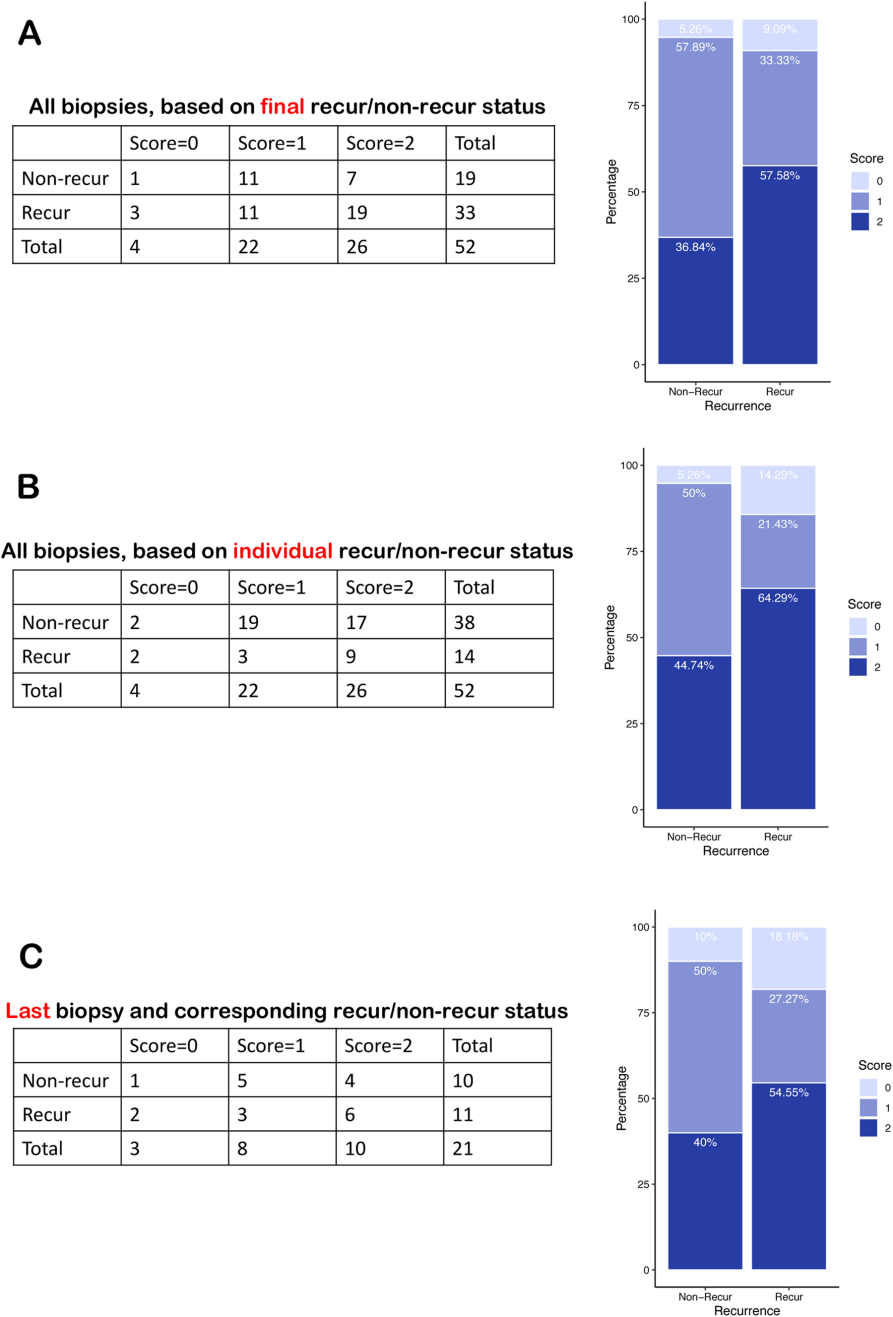
β-catenin score distribution across patient status shows an association between β-catenin nuclear localization and disease recurrence. (**A**) A continency table classifying all of the data from a patient based on the final status (recurrent or non-recurrent) shows that the majority of non-recurrent patient samples have membranous β-catenin (57.89%), while the majority of recurrent patients have nuclear β-catenin (57.58%). *p* = 0.233 by Fisher’s exact test. (**B**) Summarizing the data for recurrent and non-recurrent patients independently, irrespective of final patient status, shows that samples classified as non-recurrent are more likely to have membranous (50%) than nuclear β-catenin (44.74%), while samples showing recurrence have more nuclear β-catenin (64.29%) than any other category. *p* = 0.1209 by Fisher’s exact test. (**C**) When only the last biopsy for each patient is tallied, non-recurrent patients have mostly membranous β-catenin (50%), while recurrent patients have mostly nuclear β-catenin (54.55%). *p* = 0.6049 by Fisher’s exact test.

The relationship of β-catenin localization to disease recurrence was analyzed in three different ways. Samples were first categorized as either recurrent or non-recurrent based on the final status of the patient; if the patient was classified as ‘recurrent’, then all the biopsies for that particular patient were counted as recurrent. The number of recurrent and non-recurrent samples were then distributed by β-catenin score (Fig. 5A). In this categorization, the majority of non-recurrent samples had membranous β-catenin (57.89%), while the majority of recurrent patients showed weak nuclear staining (57.58%). We then stratified the samples based on the classification of each sample independent of the patient’s ultimate classification; because most patients did not show recurrence until their penultimate or final biopsy, the pool of non-recurrent samples was larger than the recurrent ones (38 vs. 14; Fig. 5B). Nonetheless, the pattern was similar to that obtained through classification by subject outcome, with membranous staining the most predominant subset in the non-recurrent samples (50% for membranous vs. 5.26% for absent and 44.74% for nuclear), while the recurrent group had more nuclear staining (64.29%) than either absent (14.29%) or membranous staining (21.43%). We also considered only the final biopsy from each patient and determined the staining distribution for both the recurrent and non-recurrent groups (Fig. 5C). Membranous was the most common of the three scores in the non-recurrent group (50% for 1, vs. 10% for 0 and 40% for 2), while in the recurrent group nuclear was the most common score (54.55% for 2 vs. 18.18% for 0 and 27.27% for 1). Thus, three independent analyses yielded similar findings; mainly, that recurrent samples were more likely to have nuclear β-catenin staining than non-recurrent samples, which showed predominantly membranous staining.

To determine whether nuclear localization of β-catenin could be used as an indicator of disease recurrence, a binomial logistic regression was fitted to study the importance of β-catenin score and years-to-endpoint in predicting final recurrence status. We used a time cutoff of 10 years for the analysis in order to eliminate outliers (Supplemental Table 3). For this regression, RecurStatus (0 or 1) = a0 + a1*S1 + a2*S2 + a3*time. The categorical pathological score is represented by two variables: S1 and S2, where (S1, S2) = (0,0) for score = 0; (S1, S2) = (1,0) for score = 1 and (S1, S2) = (0,1) for score = 2. Time in years is used for the time variable, and the coefficient (a0, a1, etc.) is estimated by fitting the regression model. The y (recurStatus) is the binary recurrence status, where 1 is for recur and 0 for non-recur. The output of the regression is shown in Supplemental Fig. 1.

ANOVA test on the regression coefficients shows the *p* value of β-catenin score to be 0.017 and years-to-endpoint to be 0.00063. These indicate the strong association of β-catenin score and years-to-endpoint in the prediction of final recurrence status. Thus, the appearance of nuclear β-catenin in serial biopsies positively correlates with recurrence of PSC.

**Figure 6:**
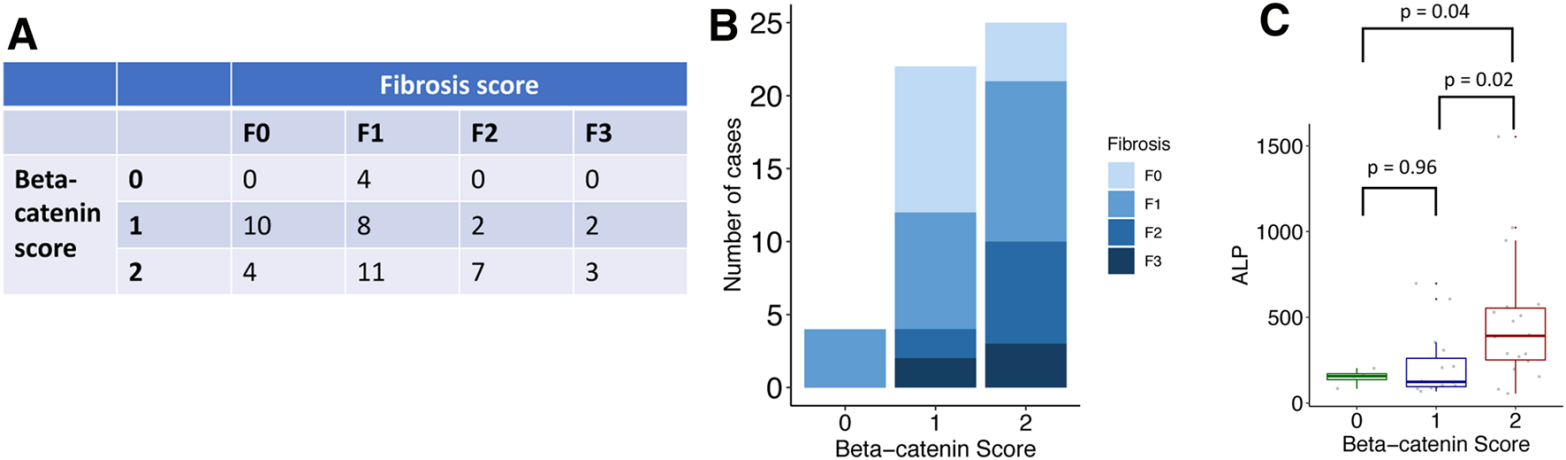
Nuclear β-catenin is associated with a nonsignificant increase in portal fibrosis and higher levels of serum ALP. (**A**) A continency table summarizing the distribution of fibrosis scores across the three categories of β-catenin staining. (**B**) Summarizing the data shows that 45% of samples with membranous β-catenin have no fibrosis, while the majority (84%) of samples with nuclear β-catenin have at least mild fibrosis. *p* = 0.108 by Fisher’s exact test. (**C**) Box and whisker plot shows a significant association between β-catenin localization and levels of serum ALP.

We then correlated the β-catenin IHC scores to two clinical parameters of biliary injury, serum ALP and fibrosis score, in order to determine the relationship between these variables. Fibrosis scores were determined from pathology reports assessing the histology of the periportal region and were categorized as follows: none (0); mild (1); moderate (2); severe (3). The fibrosis scores were then distributed by β-catenin scores (Fig. 6A). Figure 6B shows that samples with nuclear β-catenin tend to have more fibrosis (84% have mild to severe fibrosis, compared to only 54% of samples with membranous β-catenin); however, the association was not significant. We next plotted ALP levels against β-catenin score. Of non-recurring patients, 11/19 biopsies had corresponding ALP, while there were ALP values for 26/33 of the recurring biopsies (Supplemental Table 3). Despite the limited sample size, patients with nuclear β-catenin in their biopsies had significantly higher ALP levels than patients with absent β-catenin (*p* = 0.04) or membranous β-catenin (*p* = 0.02; Fig. 6C). The close association between the two variables suggests that ALP might also be a marker of progression. Indeed ALP was shown to be a predictor of disease recurrence (p-value 0.000147 by ANOVA; Supplemental Fig. 2). Therefore, the diagnostic capability of β-catenin localization was similar to ALP in discriminating recurrent from non-recurrent patients.

## Discussion

FXR agonists, which decrease bile acid biosynthesis and increase transport, have been touted as potential treatments for cholestatic liver disease associated with accumulation of toxic bile^20–23^. In fact, the FXR agonist obeticholic acid (OCA; also known as 6-ethyl chenodeoxycholic acid (6-ECDCA) or INT-747) has been FDA approved for use in patients with primary biliary cholangitis (PBC)^24,25^. A phase II clinical trial has demonstrated that OCA also leads to a significant reduction in serum markers of liver injury and cholestasis in patients with PSC^26^. Like FXR agonism, loss of β-catenin markedly improves the cholestatic phenotype through suppression of bile acid synthesis in a mouse model of biliary injury. Therefore, we hypothesized that downregulation of β-catenin may be a possible mechanism by which the liver adapts to chronic cholestatic liver injury. Indeed, we found that β-catenin protein levels are significantly decreased in liver explants from adult PSC patients. On the other hand, in an analysis of liver biopsies post-transplant, nuclear β-catenin was more frequently associated with recurrent PSC, suggesting that activation of β-catenin may contribute to disease progression. This points to a dynamic process whereby β-catenin expression and/or activation changes during the progression of disease, indicating that it may be useful as a biomarker that could help with disease staging or prognosis.

Because of the low incidence of PSC, the difficulties in assessing cholangiocyte injury in humans, and the heterogeneity of the PSC population, the initiation and progression of PSC is not well understood^27^. Thus, animal models are often used to study pathogenesis and evaluate novel therapeutic strategies. To date, no animal model fully recapitulates all of the clinical and pathological features of PSC; however, several exhibit certain characteristics resembling PSC^28^. Bile duct ligation (BDL) is a commonly used experimental model of “toxic bile” accumulation that results in cholestasis, portal inflammation, fibrosis, and proliferation of cholangiocytes^29^. Although BDL more closely resembles a model of acute biliary obstruction than sclerosing biliary disease, it is nonetheless useful in elucidating the pathways involved in the progression of cholestatic liver injury and peribiliary fibrosis. Mice with a disruption of the *Mdr2* gene (Mdr2 KO) spontaneously develop sclerosing cholangitis, characterized by pericholangitis, atypical ductular proliferation, and onionskin-type periductal fibrosis, which mirrors some of the key features of human PSC^30,31^. The mechanism of liver injury is thought to be defective biliary phospholipid secretion, which results in production of “toxic bile” containing an increase in hydrophobic bile acids^30^. This in turn causes cholangiocyte injury leading to production of pro-fibrogenic cytokines. However, the role of Mdr3 (the human orthologue of mouse Mdr2) variants in the pathogenesis of human PSC is still unclear^32^. Since no single animal model exhibits all attributes of PSC, it may be relevant to study multiple models as complementary approaches to evaluate and validate the contribution of cell signaling pathways.

In the mouse BDL model, we found that loss or inhibition of β-catenin led to decreased hepatobiliary injury^13^. This is due to suppression of bile acid synthesis which is enhanced by loss of β-catenin, providing evidence that inhibiting this pathway during cholestasis may alleviate injury and progression of disease. Interestingly, however, β-catenin suppression in the Mdr2 KO led to exacerbation of hepatic and biliary injury^33^. Since β-catenin plays an important role in the regenerative response, it is likely that the chronic injury resulting from loss of Mdr2 is worsened when the liver is unable to repair itself, leading to a more severe phenotype. This dichotomy is recapitulated in our explant studies. The majority of explanted liver tissue from end-stage PSC patients had decreased β-catenin coincident with decreased Cyp7a1 expression. The loss of β-catenin in these livers is global, affecting both β-catenin at the membrane surface and active β-catenin in the pericentral region, whereas loss of Cyp7a1 would most likely occur primarily in the pericentral region due to its regulation by β-catenin. As a consequence of decreased bile acid synthesis, hepatic bile acid levels were equivalent in both PSC and NL. Several other studies have shown no difference in biliary bile acid concentrations between PSC patients and controls, supporting this finding and suggesting that β-catenin suppression may be beneficial in preventing overproduction of harmful bile acids^34,35^. On the other hand, loss of β-catenin may also impede critical processes such as regeneration during ongoing injury, which may ultimately contribute to liver failure. In fact, the importance of β-catenin in prevention of liver failure was demonstrated in a study showing that increased nuclear β-catenin and hepatocyte proliferation was associated with spontaneous regeneration after acetaminophen overdose, alleviating the requirement for liver transplant^36^. Because the livers in our first cohort were explanted, it means that all attempts to compensate for injury had failed, and thus we were unable to address whether loss of β-catenin alleviates or exacerbates end-stage liver disease in PSC.

However, analysis of the biopsies allows us to look at β-catenin activation and expression as a function of time, which can potentially inform our interpretation of the explants. Notably, in our longitudinal studies of liver biopsies we found that activation of β-catenin (as defined by nuclear translocation) appears to predict PSC recurrence after transplant. It is not yet clear what role, if any, β-catenin signaling plays in a heterogeneous disease like PSC. However, one hypothesis supported by the biopsy data is that early activation of β-catenin in post-transplant livers might exacerbate disease progression through as-yet unknown mechanisms, while membranous β-catenin is indicative of a more mild phenotype. In light of these findings, the notable loss of β-catenin in the explanted livers indicates that in later stages of the disease, downregulating β-catenin expression may be a compensatory response by the liver to ameliorate chronic cholestatic disease.

Our findings are also in line with previous observations that active β-catenin is associated with cholestasis in HCC^37^. Whether β-catenin activation results in increased expression of Cyp7a1 or increased transcription of some other unidentified target is not known. Although evidence from mouse models suggests that this phenomenon may be attributable to increased bile acid synthesis, we were unable to verify that biopsies with nuclear β-catenin had more Cyp7a1 due to sample limitations. It should also be noted that β-catenin-driven HCC arises because of genetic mutations in members of the Wnt/β-catenin signaling pathway that result in stabilization of β-catenin. In this study, we do not propose that β-catenin translocates to the nucleus because it is mutated, as in HCC, but rather that it is activated through canonical Wnt signaling or other physiological mechanisms. Indeed, we and others have shown that several Wnts are upregulated in mouse models of cholestasis, resulting in activation of hepatocyte β-catenin^38,39^.

Interestingly, 1 recurrent patient (4848) and one non-recurrent patient (1826) showed an absence of β-catenin in their final biopsy, despite having either nuclear or membranous staining in their previous biopsies. This loss may indicate a tendency to lose β-catenin expression during the later stages of disease as observed in the explanted livers; however, because the sample size is small, further studies with larger patient populations would be needed to strengthen this finding. Further confounding interpretation is the difficulty of detecting nuclear β-catenin by immunohistochemistry, as previously documented by us and others^16,37,40,41^. Thus, it is possible that we may have underestimated the number of β-catenin-positive hepatocyte nuclei in both recurrent and non-recurrent groups. Ideally, identification of a reliable marker of β-catenin activation that is specific for cholestatic liver disease would allow for a more accurate assessment of clinical specimens.

Both animal studies and the human studies here indicate that loss of β-catenin is protective against cholestatic liver injury and may be a way that the liver adapts to chronic cholestatic injury as a natural evolution of disease. The simultaneous downregulation of Cyp7a1 indicates a possible interaction of the Wnt/β-catenin pathway with nuclear receptors such as FXR that regulate bile acid synthesis, and although this seems to be the case in the BDL mouse model, the absence of SHP upregulation and lack of hepatic bile acid accumulation in the PSC explants suggest alternative or redundant mechanisms. Conversely, increased expression or activation of β-catenin is an indicator of worse prognosis. β-catenin localization also closely correlated with ALP level, which is also an independent predictor of recurrence in our study. Our studies revealed that the diagnostic capability of β-catenin localization is similar to that of ALP. While fibrosis may not be an accurate predictor of disease recurrence, it may be desirable to perform β-catenin IHC on biopsies taken during post-transplant follow-up in order to support a prediction of recurrence in conjunction with ALP levels. Extending this concept, it would be interesting to correlate β-catenin localization with patient response to therapeutic treatment such as OCA or ursodeoxycholic acid (UDCA), which would indicate whether β-catenin activation could be altered or even reversed. Unfortunately, data on what medication a patient received, if any, was unavailable, and thus we were not able to determine if there was any relationship between the variables.

It it tempting to speculate that β-catenin is not only a biomarker for risk stratification but also a potential causative factor in PSC progression. However, as animal studies have demonstrated, β-catenin is pleiotropic and can likely have multiple functions during cholestatic liver disease, including reducing bile acid synthesis, inducing regeneration and hepatocyte proliferation, and reducing oxidative stress. Therefore, caution should be exercised when interpreting the results from these preliminary findings, especially in light of the limited size of the cohorts. Additional analyses with larger numbers of patient cases will be needed to substantiate the findings in this retrospective study and also address the mechanism by which the liver may be suppressing β-catenin during end-stage cholestatic liver disease. Nonetheless, these studies are proof-of-concept that β-catenin expression and localization may be a useful prognostic tool for PSC.

## Supplementary Information

Supplementary Information 1.

Supplementary Information 2.
