# Supplementary material for: Changes in beta-catenin expression and activation during progression of primary sclerosing cholangitis predict disease recurrence
Source: Sci Rep. 2022 Jan 7;12:206. doi: 10.1038/s41598-021-04358-6 (PMC8741932; doi:10.1038/s41598-021-04358-6)

Beta-catenin expression and activation in primary sclerosing cholangitis: pathogenic and diagnostic implications

Mary Ayers[1], Silvia Liu[2,3], Aatur D. Singhi[2,3], Karis Kosar[2], Pamela Cornuet[2], *Kari Nejak-Bowen[2,3]

[1]Children's Hospital of Pittsburgh, Pittsburgh, PA; [2]Department of Pathology, University of Pittsburgh, Pittsburgh, PA; [3]Pittsburgh Liver Research Center, University of Pittsburgh, Pittsburgh, PA

Supplemental Figure 1

```
Call:
glm(formula = RecurrenceBinary ~ Pathology.Score + Years.to.tx,
    family = binomial(link = "logit"), data = resSub)

Deviance Residuals:
    Min       1Q   Median       3Q      Max
-1.7471  -0.6635  -0.2679   0.5443   2.1876

Coefficients:
                  Estimate Std. Error z value Pr(>|z|)
(Intercept)        -5.9207     2.2099  -2.679  0.00738 **
Pathology.ScoreS1   1.6689     1.6073   1.038  0.29911
Pathology.ScoreS2   3.8390     1.8254   2.103  0.03546 *
Years.to.tx         0.6199     0.2177   2.847  0.00441 **
---
Signif. codes:  0 ‘***’ 0.001 ‘**’ 0.01 ‘*’ 0.05 ‘.’ 0.1 ‘ ’ 1

(Dispersion parameter for binomial family taken to be 1)

    Null deviance: 55.433  on 46  degrees of freedom
Residual deviance: 40.144  on 43  degrees of freedom
AIC: 48.144

Number of Fisher Scoring iterations: 5
```

Supplemental Figure 2

```
Call:
glm(formula = Recurrence ~ ALP, family = binomial(link = "logit"),
    data = data)

Deviance Residuals:
    Min       1Q   Median       3Q      Max
-1.5824  -0.6088  -0.4307   0.3029   2.0110

Coefficients:
             Estimate Std. Error z value Pr(>|z|)
(Intercept) -2.882769   0.876780  -3.288  0.00101 **
ALP          0.006267   0.002270   2.760  0.00577 **
---
Signif. codes:  0 ‘***’ 0.001 ‘**’ 0.01 ‘*’ 0.05 ‘.’ 0.1 ‘ ’ 1

(Dispersion parameter for binomial family taken to be 1)

    Null deviance: 46.626  on 36  degrees of freedom
Residual deviance: 32.219  on 35  degrees of freedom
  (15 observations deleted due to missingness)
AIC: 36.219

Number of Fisher Scoring iterations: 5
```

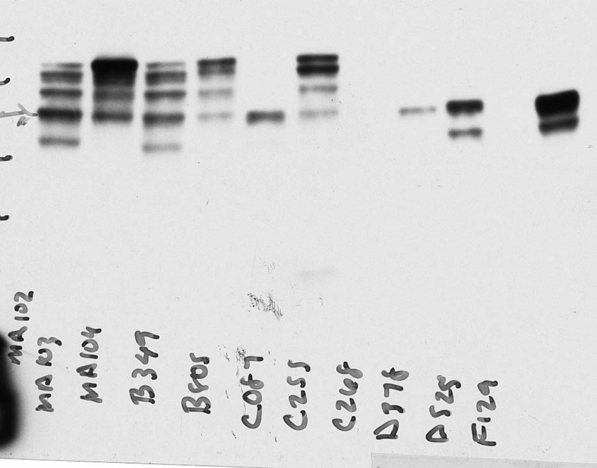
MA102
MA103
MA104
B347
C255
C268
D525
F129

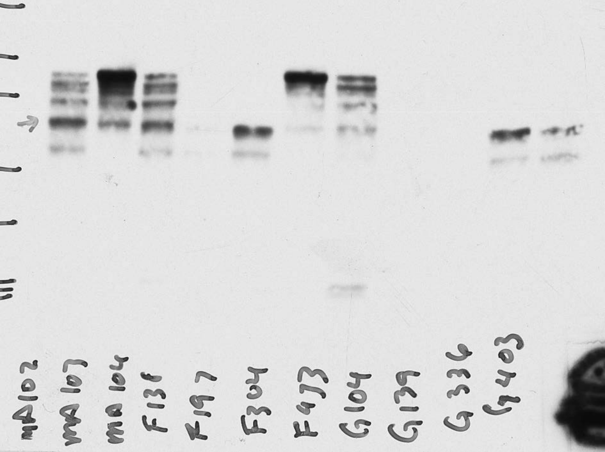
mA102
mA103
mA104
F138
F197
F304
F473
G104
G139
G336
G403

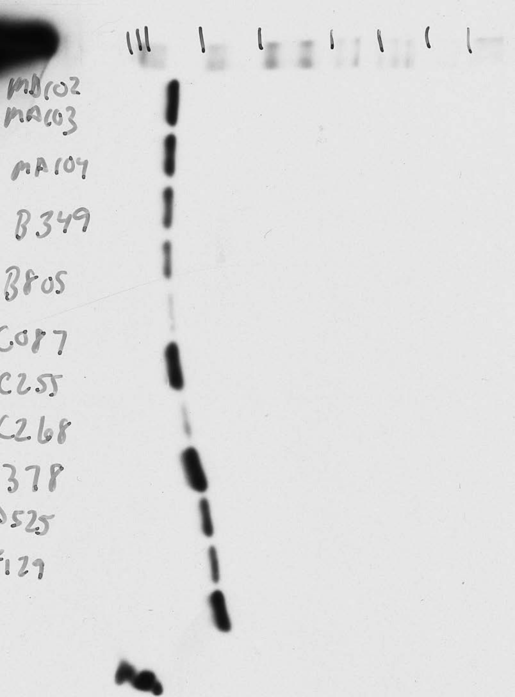

mA103
mA104
B349
B805
C087
C255
C268
378
525
129

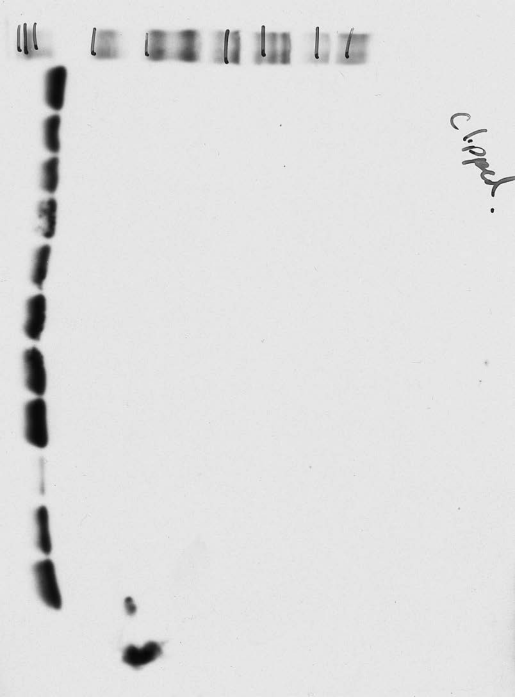

Supplement: Supplementary file 2 — Supplementary Information 2. [file 41598_2021_4358_MOESM2_ESM.pdf]
